# Analysis of the pattern of suprahyoid muscle activity during pharyngeal swallowing of foods by healthy young subjects

**DOI:** 10.3109/03091901003646096

**Published:** 2010-04-13

**Authors:** I. Ashida, H. Iwamori, S.-Y. Kawakami, Y. Miyaoka, A. Murayama

**Affiliations:** Department of Health and Nutrition, Niigata University of Health and Welfare, School of Health Sciences, 1398 Shimami-cho, Niigata 950-3198, Japan

**Keywords:** Muscle activity pattern, Suprahyoid muscle, Swallowing, Texture, Normal subject

## Abstract

We previously developed the T_P_ technique to discriminate between the activity patterns of skeletal muscles. In this study we aim to identify the T_P_ value(s) that can be used to sensitively evaluate the activity patterns of the suprahyoid (SH) muscles during swallowing. We also analyse the effect of food textural properties on the activity patterns of the SH muscle during oral and pharyngeal swallowing. Three test foods consisting of 3%, 6% and 9% of a thickening agent, Mousse-up (MU) were prepared. Their textural properties differed significantly. Swallowing of 9% MU involved a significantly longer average duration than 3% MU. The average T_50_ value for 6% MU was significantly larger than that for 3% MU. However, the average T_2_0 and T_80_ values of the test foods did not differ. Thus, the T_50_ value is particularly suitable for evaluating SH muscle swallowing patterns. Moreover, test foods that vary in their textural properties elicit different durations and patterns of SH muscle activity.

## 1. Introduction

Along with the use of imaging tools such as videofluorography, ultrasonography and fibroscopy, analysis of swallowing-related muscle activity in basic research and in the clinic has helped to improve our understanding of the mechanisms involved in swallowing [1,2]. Most studies have measured conventional parameters such as the durations and amplitudes of muscle discharge, which have been expressed as peak values and root mean square values. In contrast, our group has analysed the activity patterns of the swallowing-related muscles [3–5]. Moreover, we previously developed the T_P_ technique to discriminate between the activity patterns of skeletal muscles, and have used this technique to analyse the activities of the chewing and swallowing-related muscles [3–6]. We found that the taste stimuli provided by various test foods alter the activity patterns of the suprahyoid (SH) muscles [4,5]. We also observed that rheological properties of test foods such as texture affect the activity patterns of the masseter muscles [6]. However, we have not yet examined whether test food texture also affects the patterns of SH muscle activity, although it is known that textural changes alter the durations and amplitudes of SH muscle activity (e.g. [7]). Here, we used the T_P_ technique to test whether the texture of test foods affects the patterns of swallowing-related SH muscle activity.

In our T_P_ technique we usually calculate nine T_P_ values from T_10_ to T_90_. However, it remains unclear whether all of these T_P_ values are equally informative in terms of evaluating muscle activity patterns. Consequently, we here also investigated whether particular T_P_ values are more informative than other values with regard to analysing the activity pattern of the SH muscles.

## 2. Materials and methods

### 2.1. Subjects

Five healthy subjects (two males and three females, 18–19 years old) participated in the present study. The aims, methods and safety of the experiment were explained to the subjects individually. Informed consent was obtained from all subjects.

### 2.2. Test foods

A thickening agent, Mousse-up (MU; Nisshin Science, Yokohama, Japan) was used as a test food in this study. Three test foods that were designated as 3%, 6% and 9% MUs were prepared by dissolving 3.0, 6.0 and 9.0 g MU in 100 ml of distilled water, respectively. Visual observations and feeling the foods with the hand indicated that, texturally, the three foods resembled tomato juice, albumen, and mayonnaise, respectively. All three test foods were placed into needle-less plastic syringes (10 ml per syringe). By using a thermostat bath, the syringes were kept at a temperature of 378C, which is roughly equivalent to the oral temperature.

### 2.3. Textural properties

Three characteristic mechanical values: hardness (force required to penetrate a food with the molar teeth), adhesiveness (force required to remove a food that adheres to the mouth surface), and cohesiveness (degree of deformation of a food until rupture) were adopted as textural properties of test foods, because they have previously been defined as basic and typical properties of foods in the food sciences [8]. The textural properties of the 3%, 6% and 9% MU foods were measured by using a texture profile unit (TPU-2S, Yamaden, Tokyo, Japan) at room temperature (about 25°C). Each sample was placed into a stainless steel container (40 mm in diameter and 15 mm deep) and then compressed twice by a 20-mm diameter plunger on the textural profile unit at a speed of 10 mm s^−1^ with a clearance of 5.0 mm. The measurements were repeated 10 times for each sample. The data collected are presented as means ± standard deviations (SD).

### 2.4. Recording

**Figure 1:**
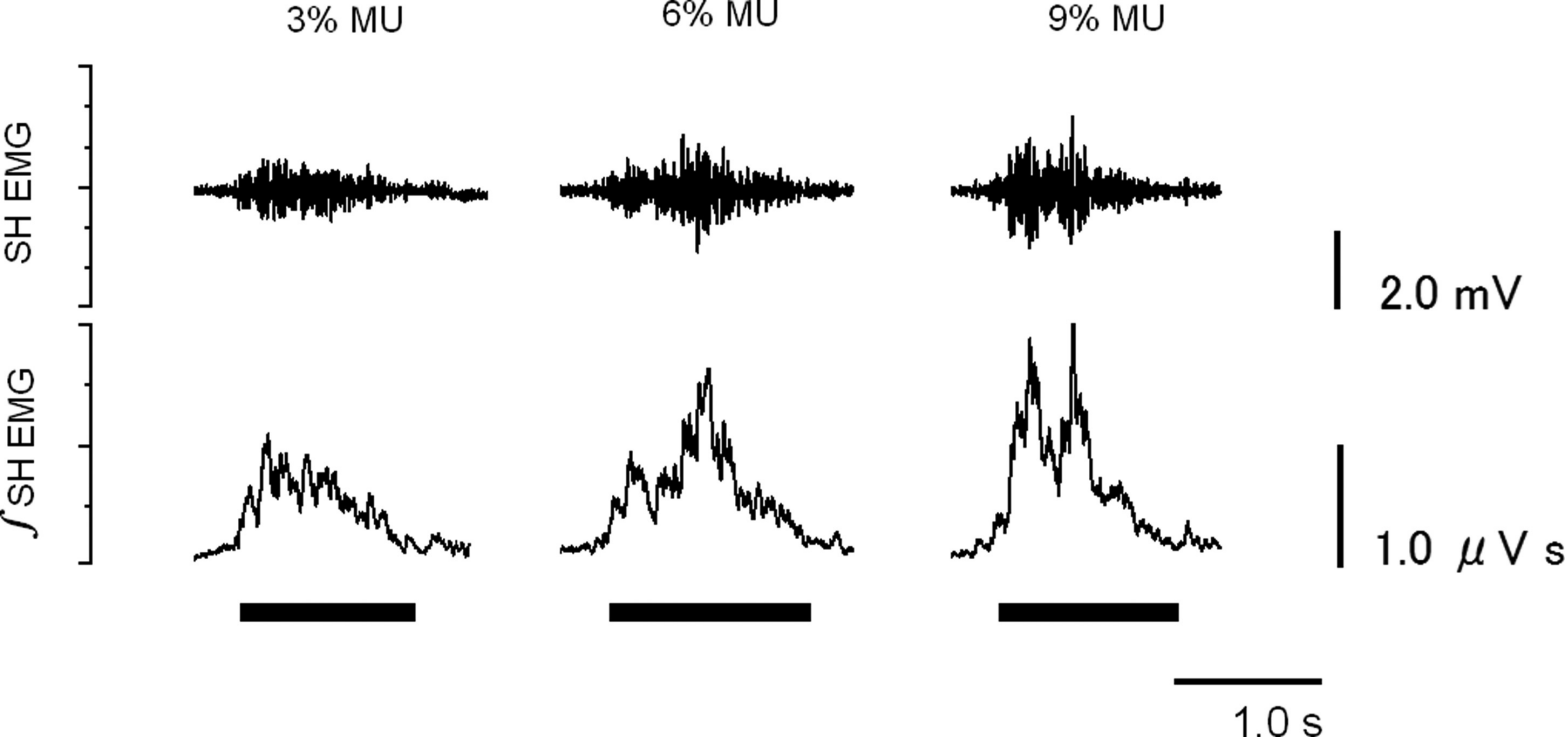
Sample data of suprahyoid muscle activity during the swallowing of the three test foods. The two traces depict electromyograms recorded from the suprahyoid muscles (∫SH EMG) and their integration (∫SH EMG) during the swallowing of the three test foods. The thick horizontal lines under ∫SH EMG indicate the periods from the start to the end of swallowing.

Surface electromyograms (sEMG) of the SH muscles were recorded by using a pair of adhesive electrodes (Blue Sensor, Ambu, Glen Burnie, MD, USA). The electrodes were adhered to the skin under the chin on both sides of the midline. In addition, a reference electrode was affixed to the right earlobe by an ear-clip. Signals of the sEMG were amplified, full-rectified, filtered (with a passband of 10–5000 Hz), and integrated (time constant = 0.03 s) to obtain ∫SH EMG waveforms (see the lower trace of figure 1), which indicate the force exerted by the muscles. The data were stored (digital sampling rate, 10 kHz) on a data recorder (ADInstruments, PowerLab/8sp, Bella Vista, Australia) for later analysis.

### 2.5. Procedures

After setting up a room for the sEMG recording, each subject was instructed to sit comfortably on a chair in the room, which was at room temperature (about 25°C). The subject was asked to open his or her mouth and protrude the tongue as far as possible; the data obtained from this test were used to analyse the swallowing data later. The instructions to the subjects in every trial were as follows: (1) open the mouth slightly and hold that position for about 5 s, (2) accept 3 ml of the food poured from a syringe onto the tongue, (3) close the mouth, (4) swallow the food without chewing when a command light is turned on, and (5) rinse the mouth with water if necessary. The trials were separated by approximately 2 minutes.

### 2.6. Data analysis

Since the subjects varied markedly in terms of the amplitudinal changes in the SH muscles, the amplitude of the SH muscles during swallowing was standardized by being divided by the amplitude recorded while the tongue was being protruded as far as possible in each experimental session. The three parameters measured in this study were: (1) the duration of pharyngeal swallowing, approximated by the duration of SH muscle activity, as indicated by previous investigations of swallowing motions with a VF and/or piezoelectric sensor [9,10]; (2) the cumulative amplitude of SH muscle activity during pharyngeal swallowing; and (3) the ratio of the cumulative amplitude relative to the duration of pharyngeal swallowing.

The T_P_ technique was developed to quantitatively evaluate the activity patterns of the sEMGs [3–6]. Briefly, in this technique, (1) a cumulative sEMG value during swallowing (i.e. ‘final cumulative value') is calculated; (2) the cumulative value is divided into 10 (in the present study, for higher temporal resolution) equal sections; (3) the 10 sectioned values are projected onto the horizontal (temporal) axis; and (4) a T_P_ (T_10_, T_20_, …, T_100_) value is standardized as the ratio of each of the projected values to the swallowing duration. In the T_P_ technique, each T_P_ value designates the relative location of the sEMG activity on the temporal axis. For example, a T_50_ value would indicate the standardized time when the cumulative sEMG activity reaches half of the final cumulative value. A smaller T_P_ value would suggest a negatively skewed (skewed to right) distribution for the enveloped curve of the integrated sEMG activity.

### 2.7. Statistical analysis

One-way analysis of variance (ANOVA) followed by Tukey's multiple-comparison test was used to determine whether the three test foods varied in terms of their hardness, adhesiveness and cohesiveness, the three sEMG parameters (i.e. duration, cumulative amplitude and ratio of the cumulative amplitude relative to the duration), and the T_P_ values. The level of statistical significance was set at *p* < 0.05.

## 3. Results

**Figure 2:**
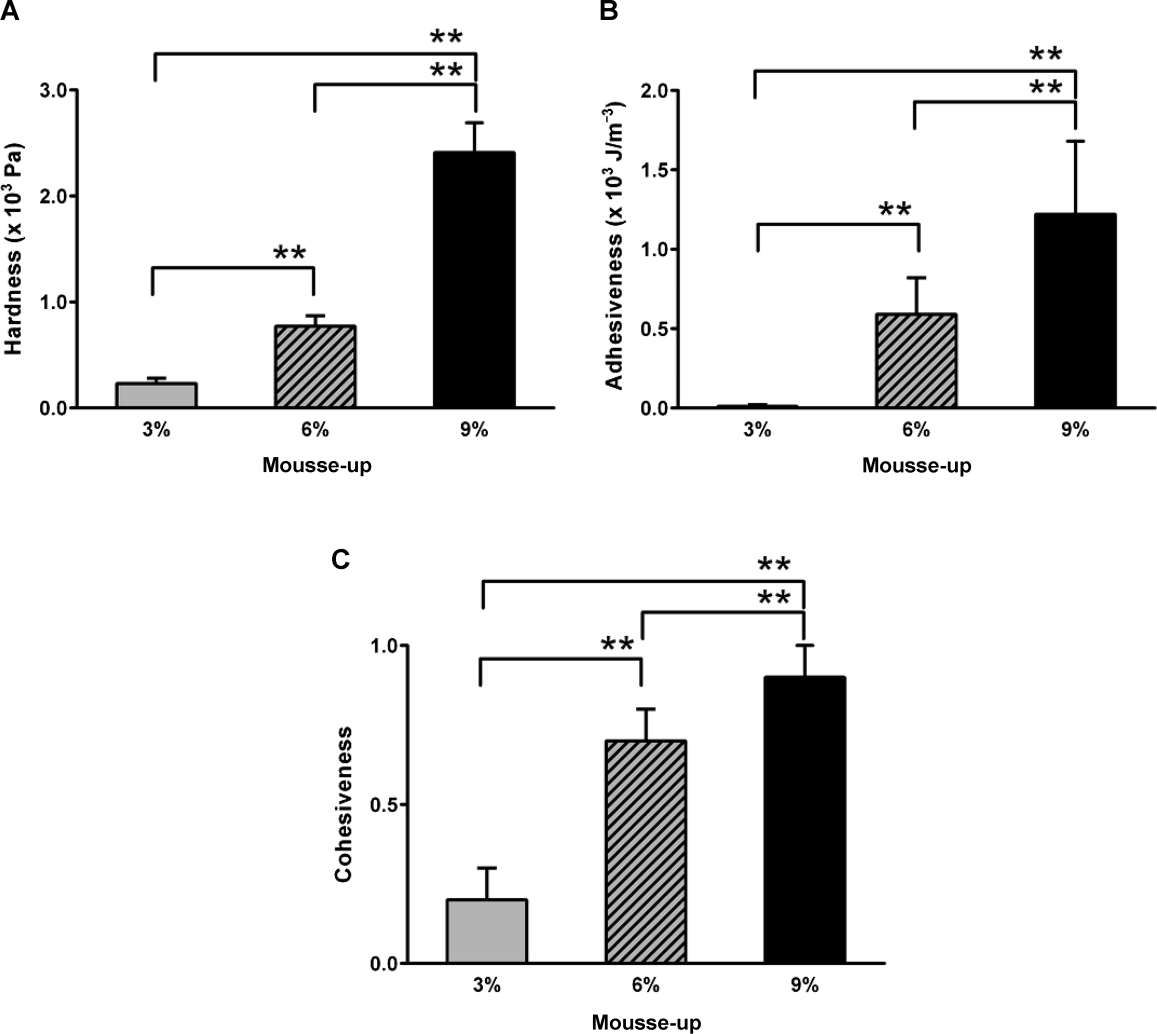
Hardness (A), adhesiveness (B) and cohesiveness (C) of the three test foods composed of 3%, 6% and 9% of the thickening agent Mousse-up. The values indicate means plus standard error of the mean. See the text for details. *p = 0.05, **p = 0.01.

Figure 2 shows the average values of the three test foods in terms of the three textural properties that were measured, namely hardness, adhesiveness and cohesiveness. Higher concentrations of MU increased all three properties. The test food that contained the least MU (3% MU) had the lowest values for all three textural properties. One-way ANOVA for the three textural properties revealed that the three test foods differed significantly in terms of all three textural properties (*p* = 0.01). Tukey's multiple-comparison tests also revealed that there were nine specific differences in the test foods in terms of these three properties of (*p* = 0.01; figure 2).

Figure 1 depicts sample data of SH muscle activity during the swallowing of the three test foods. Precise observation of the three RSH EMG for MU reveals that increasing the concentration of MU from 3% to 9% augmented SH muscle activity but did not noticeably alter its duration. It was also observed that increasing the concentration of MU changed the SH muscle activity patterns, as 3% MU generated a negatively skewed activity pattern, 6% MU induced a more symmetrical activity pattern, and 9% MU produced an activity pattern with two peaks.

**Figure 3:**
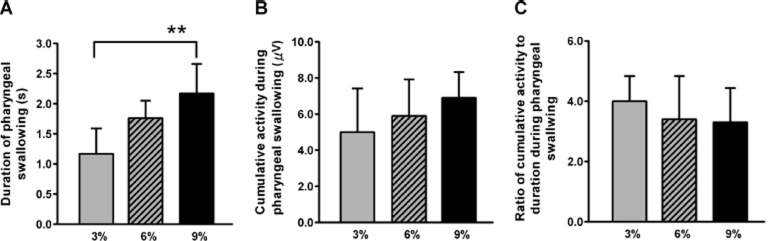
Changes in (A) duration, (B) cumulative activity of the suprahyoid muscles, and (C) ratios of the cumulative activity relative to the duration, during the swallowing of the three test foods. These three parameters were measured from electromyograms of the suprahyoid muscles that were recorded during the swallowing of three test foods whose concentrations of MU were 3%, 6% and 9%, respectively. The values indicate means plus standard error of the mean. *p = 0.05, **p = 0.01. See the text for details.

Figure 3 indicates the results of our statistical analysis of SH muscle activity during the swallowing of the three test foods. Compared to the swallowing of 3% MU, the average durations involved in swallowing 6% and 9% MU were 118.6% and 146.2%, respectively, and one-way ANOVA detected significant differences between the three durations (*p* = 0.01; figure 3A). Tukey's multiple-comparison tests followed by ANOVA revealed there were specific differences in duration between 3% MU and 9% MU (*p* = 0.01). The SH muscle activity tended to increase in a step-wise fashion as the concentration of MU changed from 3% to 9% (figure 3B), but one-way ANOVA found no significant differences between these activity values. The ratios of SH muscle activity to duration tended to decrease in a step-wise fashion as the concentration of MU changed from 3% to 9% (figure 3C), but one-way ANOVA did not detect significant differences between these ratios.

**Figure 4:**
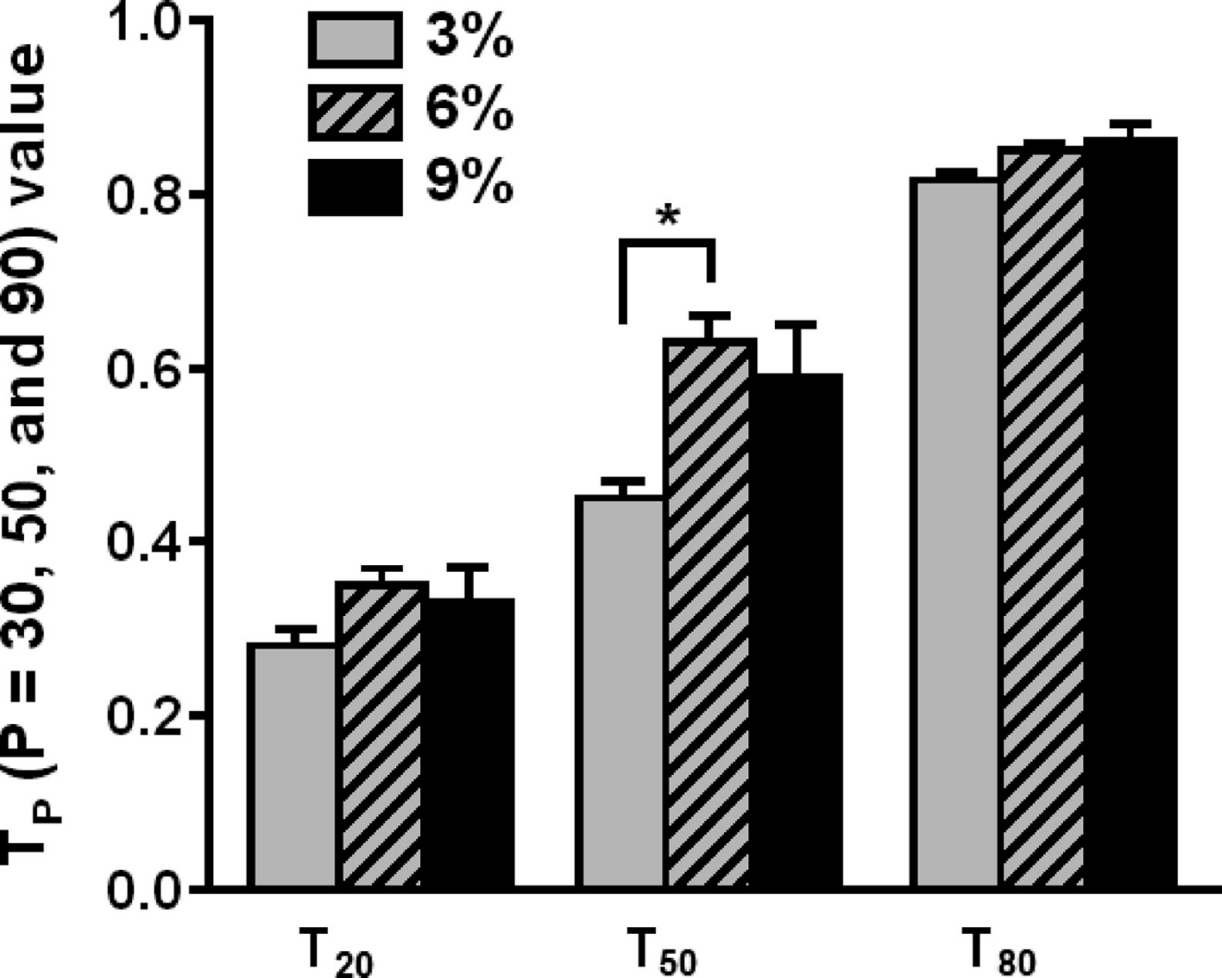
Changes in TP values during the swallowing of the three test foods. The three sets of graphs indicate the T_20_, T_50_ and T_80_ values, which were measured from the electromyograms of the suprahyoid muscles that were recorded during the swallowing of three test foods whose concentrations of MU were 3%, 6% or 9%. The values indicate means plus standard error of the mean. *p = 0.05. See the text for details.

Figure 4 extracts three T_P_ values (T_20_, T_50_ and T_80_) from the 10 that were calculated from the SH muscle activities recorded during the swallowing of the three foods. The average T_20_ values differed only slightly, ranging from 0.31 (3% MU) to 0.35 (6% MU), and statistical analysis with ANOVA did not detect any significant differences between these three T_2_0 values. In contrast, the average T_50_ values showed a much wider range, i.e. from 0.44 (3% MU) to 0.60 (6% MU), and ANOVA detected significant differences between the three T_50_ values (*p* = 0.05). Tukey's multiple-comparison test then showed that there were significant differences between 3% and 6% MU in terms of the T_50_ values (*p* = 0.05). The average T_80_ values (minimum 0.82; maximum 0.87) of the three foods also differed significantly upon ANOVA (*p* = 0.05) but Tukey's multiple-comparison test did not detect specific differences between the three test foods.

## 4. Discussion

The present study generated two major observations regarding the patterns of SH muscle activity during the swallowing of three test foods whose textural properties differed significantly. First, the T_50_ value was found to be especially informative in terms of evaluating the activity patterns of the SH muscles (figure 4). Second, T_P_ values, particularly T_50_, can effectively discriminate between the SH muscle activity patterns generated by the swallowing of these three test foods (figure 4), although texture properties of the test foods differed from those of the previous studies [3–6].

A previous study using electromyographic and manometric techniques reported the anterior and posterior tongue pressure activities during the swallowing of four test foods that had the textural properties of a liquid, a syrup, a thin paste and a thick paste; they found that the onset of the SH sEMG burst always preceded the onset of the tongue activities (figure 5 of [7]). Since the generation of tongue pressure involves the tongue and SH muscles, this observation suggests that the pattern of SH muscle activity differs in terms of pre- and post-peak durations. Since the peak time of integrated SH activity comes close to the time point where half of the final cumulative sEMG activity has passed (see §2.6), the T_50_ value of the SH muscles may correspond to a functional turning point of SH activity.

The average hardness, adhesiveness and cohesiveness values of the 3%, 6% and 9% MU test foods increased as the concentration of MU increased (figure 2). Previous studies have shown that increasing the hardness, viscosity and volume of test foods prolongs the duration of oral and pharyngeal swallowing [7, 11–15]. Similarly, a videofluoroscopic study showed that, compared to low density barium, swallowing high density barium prolongs the oral and pharyngeal transit times in healthy subjects [12]. In addition, an electromyographic and manometric study reported that the average duration of the SH muscle activity during the swallowing of high density agar fluid is longer than when low density agar is swallowed [7]. Thus, we can conclude that increasing the textural properties of our test foods is responsible for prolonging the duration of pharyngeal swallowing (figures 2 and 3A). However, increases in the textural properties did not affect the amplitude aspect evaluated by the cumulative values of SH muscle activity (figure 3B) and their functions (figure 3C). Unlike the textural properties and SH muscle activities, which increased or decreased monotonically as the concentration of MU in the test food changed, the T_P_ values (especially the T_20_ and T_50_ values; figure 4) were maximal when the MU concentration was 6%. Moreover, significant specific differences were detected between the T_50_ values of the 3% and 6% MU foods. The fact that the T_50_ value of the 3% MU food is smaller suggests that SH activity plays a relatively larger role in the early period compared to in the later period, including in terms of the generation of lingual pressure and pharyngeal transmission.

The two findings of the present study do not necessarily correspond completely with the findings we have reported in our previous studies [4–6]. These discrepancies are likely to relate to the properties that were tested (taste versus texture) and the muscles that were analysed (SH versus masseter). Additional research is needed to determine the meaning of the T_P_ values (in particular the T_50_ value) in various situations.

In conclusion, the T50 value is particularly informative for evaluating the swallowing pattern of swallowing-related (SH) muscles. Moreover, textural differences between foods affect the patterns of SH muscle activity when they are expressed by T_P_ values.
